# Cytological diagnosis of squamous cell carcinoma of renal pelvis

**DOI:** 10.4103/0970-9371.70756

**Published:** 2010-04

**Authors:** Rubi Bindra, Saurav Gupta, Neelam Gupta

**Affiliations:** Department of Pathology, I.G.M.C, Shimla, India

Sir,

Primary squamous cell carcinoma of the renal collecting system is rare. It is more frequently reported in the bladder and male urethra than in pelvis.[1] The incidence of squamous cell carcinoma among malignant renal tumors ranges widely from 0.5% to 0.8%.[2] It commonly occurs in patients with long standing renal calculi. Fine-needle aspiration cytology (FNAC) has become an important tool in diagnosing squamous cell carcinoma of the kidney. We would like to share our experience in diagnosing one such case on urine cytology and FNAC.

A 50-year-old woman presented with left flank lump with pain and fever for two months. Physical examination was normal except for left-sided abdominal tenderness. Ultrasonography revealed hydronephrosis. Intravenous pyelography showed a functioning kidney with a slight left hydronephrosis and multiple calculi in the lower calyx. The patient was diagnosed to have hydronephrosis with calculi.

**Figure 1:**
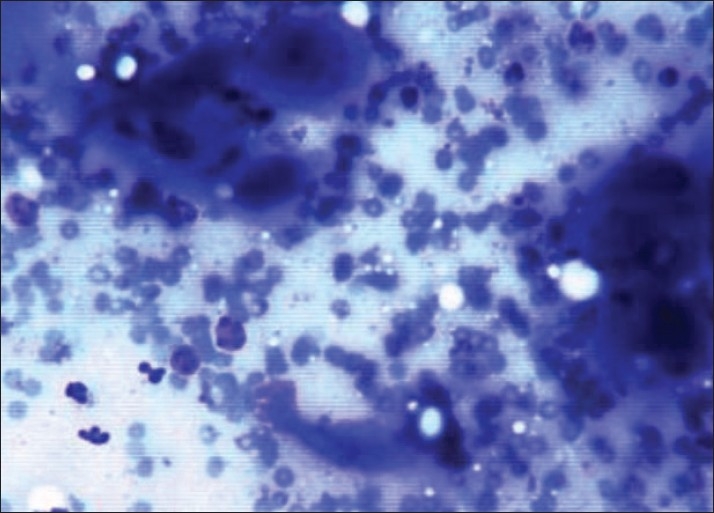
Fine-needle aspiration cytology smear shows pleomorphic squamous cells in a hemorrhagic background (Giemsa stain, ×400)

We received four mL of straw colored urine, which was centrifuged, and smears were made from the sediment. On microscopic examination, urinary cytology was positive for malignant cells. Cytomorphological feature of squamous cell carcinoma was suggested [Figure 1]. Following this, ultrasound-guided FNAC of the lump was performed.

**Figure 2:**
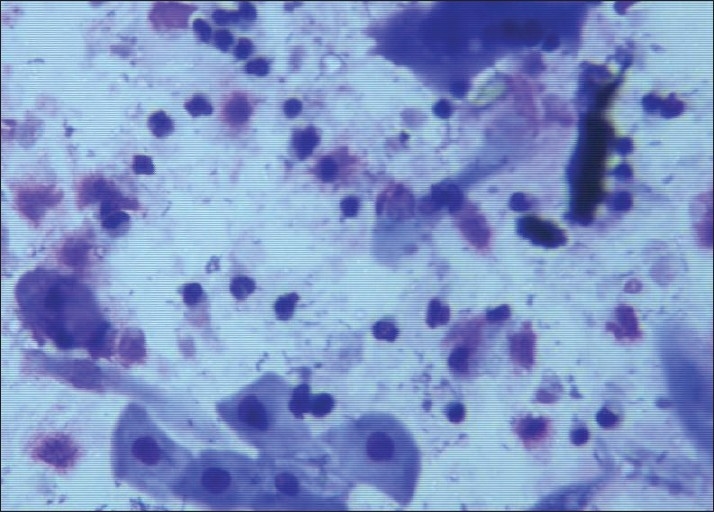
Urine smear shows benign urothelial cells and pleomorphic squamous cells (Giemsa stain, ×400)

FNAC smears showed acute inflammatory cells, a few anucleate squames, necrotic cellular material, and a few isolated and occasional groups of epithelial tumor cells. The cells showed pleomorphism, central hyperchromatic nucleus with coarse irregular chromatin, and inconspicuous nucleoli; irregular nuclear outlines and mild to moderate amount of ground-glass–like basophilic cytoplasm were also observed. Occasional tadpole cells were also seen in a hemorrhagic background [Figure 2]. Considering the above features, a diagnosis of squamous cell carcinoma was made.

Renal FNAC can produce an accurate diagnosis in most cases. FNAC when practiced in a multidisciplinary setting with direct involvement of pathologists, radiologists and clinicians is an extremely accurate, well-tolerated, relatively noninvasive, and low-risk test that obviates the needs for surgical intervention in most benign conditions and disseminated malignancy. Therefore, by taking an active role with onsite assessment of the FNAC material and discussion with radiologist colleagues, the cytopathologist can offer a FNAC service comparable to surgical pathology in sensitivity and very similar to frozen section in specificity.[3]

Squamous cell carcinoma is estimated to represent 9%–20% of all tumors of the renal pelvis. Urinary calculi are accepted as main carcinogenic risk factors for squamous cell carcinoma. Chronic irritation and infection are believed to induce infective changes in the urothelium and lead to neoplasm via metaplasia and leucoplakia.

Uretral obstruction is the main cause of the presenting symptom. Squamous cell carcinoma of upper urinary tract is generally considered as an aggressive tumor.

Treatment of choice is nephrectomy with total ureterectomy, including bladder cuff around the ureteral orifice.[4] Chemotherapy conveys little benefit and value of radiotherapy is debatable.

We have reported this case to highlight the fact that imaging techniques usually reveal only calculi and hydronephrosis, but FNAC can provide us with an accurate diagnosis of the underlying malignanacy.
